# Effects of Interventions to Prevent Work-Related Asthma, Allergy, and Other Hypersensitivity Reactions in Norwegian Salmon Industry Workers (SHInE): Protocol for a Pragmatic Allocated Intervention Trial and Related Substudies

**DOI:** 10.2196/48790

**Published:** 2023-07-19

**Authors:** Anje Christina Höper, Jorunn Kirkeleit, Marte Renate Thomassen, Kaja Irgens-Hansen, Bjørg Eli Hollund, Carl Fredrik Fagernæs, Sindre Rabben Svedahl, Thor Eirik Eriksen, Miriam Grgic, Berit Elisabeth Bang

**Affiliations:** 1 Department of Occupational and Environmental Medicine University Hospital of North Norway Tromsø Norway; 2 Department of Community Medicine Faculty of Health Sciences UiT The Arctic University of Norway Tromsø Norway; 3 Department of Occupational Medicine Haukeland University Hospital Bergen Norway; 4 Department of Global Public Health and Primary Care University of Bergen Bergen Norway; 5 Department of Occupational Medicine St. Olavs Hospital, Trondheim University Hospital Trondheim Norway; 6 Department of Public Health and Nursing Faculty of Medicine and Health Sciences Norwegian University of Science and Technology Trondheim Norway; 7 Department of Medical Biology Faculty of Health Sciences UiT The Arctic University of Norway Tromsø Norway

**Keywords:** allergy, bioaerosols, exposure-response, health promotion, hypersensitivity, occupational asthma, occupational skin disease, psychosocial work environment, salmon processing industry

## Abstract

**Background:**

Workers in the salmon processing industry have an increased risk of developing respiratory diseases and other hypersensitivity responses due to occupational exposure to bioaerosols containing fish proteins and microorganisms, and related allergens. Little is known about effective measures to reduce bioaerosol exposure and about the extent of skin complaints among workers. In addition, while identification of risk factors is a core activity in disease prevention strategies, there is increasing interest in health-promoting factors, which is an understudied area in the salmon processing industry.

**Objective:**

The overall aim of this ongoing study is to generate knowledge that can be used in tailored prevention of development or chronification of respiratory diseases, skin reactions, protein contact dermatitis, and allergy among salmon processing workers. The main objective is to identify effective methods to reduce bioaerosol exposure. Further objectives are to identify and characterize clinically relevant exposure agents, identify determinants of exposure, measure prevalence of work-related symptoms and disease, and identify health-promoting factors of the psychosocial work environment.

**Methods:**

Data are collected during field studies in 9 salmon processing plants along the Norwegian coastline. Data collection comprises exposure measurements, health examinations, and questionnaires. A wide range of laboratory analyses will be used for further analysis and characterization of exposure agents. Suitable statistical analysis will be applied to the various outcomes of this comprehensive study.

**Results:**

Data collection started in September 2021 and was anticipated to be completed by March 2023, but was delayed due to the COVID-19 pandemic. Baseline data from all 9 plants included 673 participants for the health examinations and a total of 869 personal exposure measurements. A total of 740 workers answered the study’s main questionnaire on demographics, job characteristics, lifestyle, health, and health-promoting factors. Follow-up data collection is not completed yet.

**Conclusions:**

This study will contribute to filling knowledge gaps concerning salmon workers’ work environment. This includes effective workplace measures for bioaerosol exposure reduction, increased knowledge on hypersensitivity, allergy, respiratory and dermal health, as well as health-promoting workplace factors. Together this will give a basis for improving the work environment, preventing occupational health-related diseases, and developing occupational exposure limits, which in turn will benefit employees, employers, occupational health services, researchers, clinicians, decision makers, and other stakeholders.

**Trial Registration:**

ClinicalTrials.gov NCT05039229; https://www.clinicaltrials.gov/study/NCT05039229

**International Registered Report Identifier (IRRID):**

DERR1-10.2196/48790

## Introduction

### Overview

This paper describes the protocol for an ongoing comprehensive study in the salmon industry, comprising collection of information on bioaerosol exposure and health outcomes, as well as health-promoting factors among salmon processing workers. It includes the description of an intervention trial and several substudies.

### Background and Previous Research on Salmon Industry Workers

Current knowledge is sufficient to conclude that workers in the fish processing industry have increased risk of developing respiratory diseases and other hypersensitivity responses due to occupational exposure to bioaerosols (ie, bioactive molecules, including fish allergens, enzymes, microorganisms, and endotoxins) [1-3]. We previously reported on an exposure-response relationship between total protein exposure and cross-shift lung function, as well as respiratory symptoms among exposed workers [4]. In addition, we described a case of hypersensitivity pneumonitis caused by proteins from salmon muscle [5]. Impaired respiratory status has also been reported by Douglas et al [6] and Dahlman-Høglund et al [7], and the prevalence of allergy is reported to be 2%-8% [6,8]. Avoidance or reduction of hazardous exposures at the workplace is well established as a key principle for the primary prevention of occupational diseases. For individuals who have developed occupational asthma or hypersensitivity pneumonitis, delayed diagnosis and prolonged exposure may lead to further aggravation and chronification of the disease [9,10]. Early diagnosis with identification of trigger allergens is therefore of great importance.

Hand eczema, representing either an irritant or allergic contact dermatitis, is common in occupations with prolonged use of gloves and wet work [11], and such risk factors are substantial also within salmon processing [12]. Further, contact with fish products after direct contact or deposition of bioaerosols on skin may cause development of irritant contact dermatitis and protein contact dermatitis. Nevertheless, literature concerning skin symptoms within the fish processing industry is scarce [12-14].

As described above, identifying risk factors is a core activity in disease prevention at the workplace. However, there is a growing awareness of the importance of factors influencing well-being at work [15,16]. This implies a shift in focus toward health promotion and more specifically the identification of contributing psychosocial factors. That is, the individual’s experience of health-promoting working conditions. To our knowledge, very few studies have so far examined the occurrence of such factors in the working environment in this industry [17].

### Background on Salmon Processing and Bioaerosol Exposure

Slaughtering and processing, as well as cleaning and waste handling, generate bioaerosol exposure [4,7,13]. When entering the processing plant, the salmon is stunned before it is killed by cutting the gill arches and transferred to a water tank for exsanguination. Afterward, it is degutted and cleaned of internal organs with a scraper. The salmon is individually assessed and graded according to size and quality. Further on, it is either packed whole in boxes on ice, frozen, or transported to the fileting department. During fileting, the head and backbones are removed. Filets are processed further, depending on the desired end product, for example, removal of skin and cutting to portion sizes. The removed by-products are usually gathered and used for lower-grade foods, such as animal feed or extracted oil.

During salmon processing, bioaerosols are generated along the entire production line outlined above, for example, when water beams hit the fish or surfaces with organic matter. The amount of water used is high in order to meet hygiene requirements and to ensure good workflow. Bioaerosol exposure levels of workers depend on different factors, such as the amount of water used, production activity (number of fish per time unit), distribution mode of water (eg, type and number of water nozzles, jets, spray, high vs low pressure), techniques (manual vs mechanical), type of equipment (open or closed), and shielding for water spray.

It is important to note that fish processing workers are exposed to components that are usually removed from edible parts of the fish before they reach the consumer. Therefore, the importance of specific proteins and epitopes causing allergic sensitization or inflammatory reactions in the occupational setting may be different from what we know from food allergies, where many proteins are denaturized through food preparation and digestion [18].

### Rationale of the Study

Total avoidance of exposure to bioaerosols during salmon processing is not feasible; thus, reduction of exposure is considered the best approach. This is in accordance with the recommendation from the Fifth International Fishing Industry Safety and Health Conference (IFISH5) in 2018 [19] that seafood processing activity that generates excessive bioaerosols must be reduced. To our knowledge, no intervention studies aiming at reducing bioaerosol exposure have been carried out in the salmon industry.

Control measures that aim at reducing bioaerosol generation or increasing bioaerosol elimination are interventions assumed to be efficient in decreasing bioaerosol exposure. Accordingly, the arms of the intervention study that will be described later represent different intervention categories.

Proper exposure assessment, including characterization of bioaerosols, is a prerequisite for establishing preventive measures. Lack of data on the associations between exposure levels and health outcomes has so far prohibited the establishment of occupational exposure limits. A recent European Academy of Allergy and Clinical Immunology (EAACI) position paper [2] addresses the lack of concentration-response data to set internationally accepted occupational exposure limits for airborne food allergens. Also, the progression from allergen exposure to hypersensitivity responses and development of lung diseases, contact dermatitis, and protein contact dermatitis involves a continuum of measurable molecular events, including nonspecific inflammation and immunological responses. More knowledge on mechanisms behind the exposure-induced health outcomes might provide an opportunity for future applications, such as biomarkers for early prediction of disease.

Knowledge on which indicators of health-promoting work environments are present in processing plants in the salmon industry, as well as demographic differences in the perception of health-promotion factors, is lacking. This knowledge is needed both to increase the awareness of and support future health-promoting strategies in this industry.

### Aims of the Study

The overall aim is to identify effective and feasible interventions to reduce the exposure to airborne bioaerosols, thereby preventing the development or chronification of respiratory diseases, skin diseases, and allergies among salmon processing workers. Substudies aim to identify and characterize clinically relevant exposure agents, to identify determinants of exposure, and to measure the prevalence of work-related symptoms and disease. Also, the exposure-response relationship between the exposure to individual bioactive agents in bioaerosols and the prevalence of airway symptoms, altered lung function, skin symptoms, and immunological responses indicating hypersensitivity will be investigated. Finally, substudies that aim to identify factors that contribute to a healthy psychosocial work environment in the different processing plants will be conducted.

The data collection will serve multiple stakeholders, ranging from employees, employers, and occupational health and safety personnel to researchers, clinicians, decision-makers, and authorities. Bioaerosol-reducing measures will be assessed, and the most effective approach will be recommended to the industry. In addition, data on exposure characterization, development of better diagnostic tools, follow-up of health examination and symptoms, and the perceived work environment will give new knowledge to basic, clinical, and translational research.

## Methods

### Study Setting and Study Personnel

The study population comprises salmon processing workers from 9 processing plants along the Norwegian coastline, 3 in each of the included geographical areas. The plants are situated in rural areas in western Norway (surrounding area of up to 110 km from Bergen), mid-Norway (up to 190 km from Trondheim) and northern Norway (up to 400 km from Tromsø). The processing plants vary in size, production volume, technology, and number of departments. In some plants the processing line is localized in 1 building, while in other plants the processing steps are separated and localized in several buildings. This can affect both ventilation and other technical solutions which in turn can affect generation and removal of bioaerosols. In some of the factories the processing workers tend to be specialized in specific parts of the processing line (eg, slaughtering, degutting, fileting, and packing), while in others the workers take part in all processes. Shift plans vary in terms of duration of work and breaks. A detailed description of the individual plants, job categories, and tasks will be published in a separate paper.

Study personnel for the fieldwork to gather exposure and health data comprise personnel from the university hospitals and universities of Bergen, Trondheim, and Tromsø. They are competent in occupational hygiene, medicine, nursing, biotechnology, biomedical laboratory science, or sociology, and mainly participate in fieldwork at plants within their own geographical region. Those carrying out health examinations underwent specific training according to study protocols that had been developed within the project group in collaboration with external clinical specialists and in line with national and international guidelines and recommendations. In order to minimize variability in testing techniques, individual study personnel are dedicated to 1 type of health examination whenever possible, and we aim to keep the same personnel within the geographical region and between baseline and follow-up fieldwork.

### Data

#### Overview

This study consists of an intervention trial and related substudies. It will gather extensive information about bioaerosol exposure, health, and work environment for workers in the salmon industry. Fieldwork is carried out at 2 time points that are related to the intervention study: a baseline visit (T_1_) and a follow-up visit (T_2_). The visits include exposure measurements, questionnaires, and health examinations, described in more detail below.

**Figure 1:**
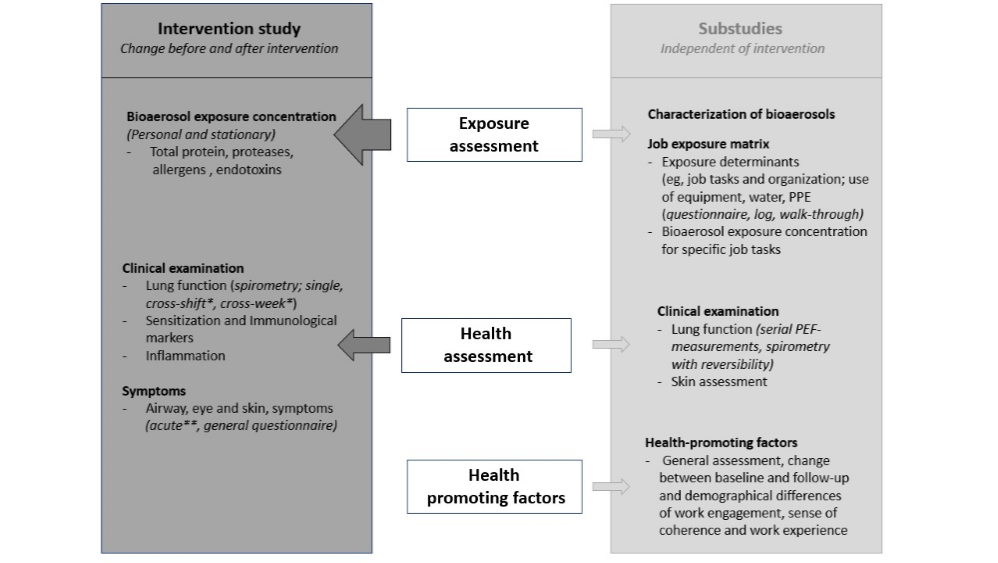
Overview of the study with its core, the intervention trial (left side), and its substudies (right side), covering the main topics (middle). Change in health-promoting factors are not part of the intervention study. *Cross-shift and cross-week examinations to be carried out with the same individual before and after shift on Monday and after shift on Thursday; **acute questionnaires to be filled out by the same individual before and after their shift, registering possible symptoms before and during their shift, as well as work tasks. PEF: peak expiratory flow; PPE: personal protective equipment.

For an overview of this comprehensive study, see Figure 1.

#### Exposure Assessment of Bioaerosols

##### Sampling Strategy

Full shift personal exposure measurements of airborne bioaerosols are collected in the workers’ breathing zone at both T_1_ and T_2_. Workers are selected based on their work tasks with the aim of covering different areas of the production process. Within each plant, we aim to include 5 job groups in the exposure assessment: workers from slaughtering, fileting, packing, the laboratory or technical department, and the central control room or administration. For the cross-week measurements, 12 workers carry a backpack with personal measuring equipment on Monday and Thursday containing 3 pumps, each connected to a filter containing a sampling head in the workers’ breathing zone. The filters are analyzed for inhalable protein, inhalable protease enzymes, fish allergens, and endotoxins. On Tuesday and Wednesday, 12 other workers carry 2 pumps whose filters are analyzed for inhalable protein, inhalable protease enzymes, and fish allergens. Workers register work tasks performed while wearing the backpack, in addition to breaks and other time away from the production area.

Stationary exposure measurements for bioaerosols are performed on Tuesday and Wednesday for a minimum of 6 hours during production. Sampling pumps are placed in plastic containers close to one of the following 3 work stations: cutting of gill arches, degutting, and trimming of filets in plants with filet departments. The filters are analyzed for inhalable protein, inhalable protease enzymes, fish allergens, and endotoxins.

Culturable bacteria and fungi are collected using a microbiological air sampler adjusted to sample 100 L and 250 L or 250 L and 500 L of air. This is done on Tuesday in close proximity to the 3 locations in each plant where the stationary bioaerosol measurements are located.

Temperature and relative humidity are registered every 15 minutes in selected areas of the processing plant throughout the week of data collection using direct reading instruments. The instruments are placed centrally in rooms with salmon production, away from sources of heat or cold.

##### Analysis of Bioaerosols

Personal and stationary bioaerosol samples are collected and analyzed for inhalable total protein (µg/m^3^), inhalable protease enzymes (ng/m^3^), fish allergens (as total dust; ng/m^3^), and endotoxins (as total dust in endotoxin units; EU/m^3^).

Inhalable protein will be quantified by bicinchoninic acid (BCA) assay [20]. Inhalable enzymes will be quantified by zymographic assay [21]. The amount of allergen will be monitored using targeted proteomic analysis using a high-throughput Q Exactive liquid chromatography–mass spectrometry (LC-MS) system [22]. Endotoxin samples will be analyzed using recombinant factor C (rFC) assay [23]).

To identify known and novel allergens, extracts from these filters will be separated by sodium dodecyl-sulfate polyacrylamide gel electrophoresis (SDS-PAGE) and subjected to immunoblotting using sera from workers with immune globulin E (IgE) reactivity to salmon (>0.1 kU/L). Antibody binding will be graded and presented in allergograms.

Identification of microorganisms to species level will be collected from area sampling on growth media. Quantification and identification of bacteria and fungi will be performed using matrix-assisted laser desorption and ionization-time of flight mass spectrometry (MALDI-TOF MS) as described by Madsen et al [24].

##### Determinants of Exposure

Determinants of exposure are factors that directly or indirectly affect the environmental concentration of the agent of interest. To register relevant information on potential determinants of exposure to bioaerosols (inhalable protein, allergen, enzymes, and endotoxins) a walk-through survey and meetings with the management during fieldwork to gather technical and process-related information will be performed. In addition, workers carrying the sampling devices will be asked about job tasks performed, number and duration of breaks, and any incidences thought to affect exposure.

#### Questionnaires

There are 2 types of self-administered questionnaires. The main questionnaire includes questions on demographics, background data regarding health and previous job history, smoking habits, department and job tasks, exposure determinants, respiratory and skin symptoms, allergies, and health-promoting factors. All employees present at work during the T_1_ and T_2_ visits are intended to be invited to answer this form. Repeated short questionnaires regarding work tasks and acute symptoms from the airways, nose, eyes, and skin will only be filled out by workers carrying out personal exposure measurements.

Questionnaires are available in Norwegian, English, Polish, Lithuanian, Slovakian, and Romanian. A Russian version of the main questionnaire is made available at T_2_. Validated questions were used whenever possible. For further information, see the “Health Assessment” section.

Distribution of main questionnaires is done either by study personnel, contact persons, or other plant staff, depending on the plant. Those who participate in health examinations usually receive their questionnaire at this time point.

#### Health-Promoting Factors

Health-promoting factors in the salmon processing industry are assessed in the main questionnaire using a compilation of standardized questionnaire-based instruments, including the Utrecht Work Engagement Scale (UWES) [25], Work-related Sense of Coherence Scale (Work-SOC) [26-28], and Work Experience Measurement Scale (WEMS) [29,30].

#### Health Assessment

Due to logistical constraints, a maximum of 99 workers at each processing plant are invited to participate in health examinations. They are prioritized based on their work tasks, with production workers being the main focus. For the T_2_ measurements, the plants are asked to aim at recruiting workers that participated in the first round when possible. Before the health examinations, participants are to fill out a form ruling out contraindications to examinations and to register their postal address so that pathological findings can be sent directly to the participant.

#### Health Examinations

##### Overview

**Figure 2:**
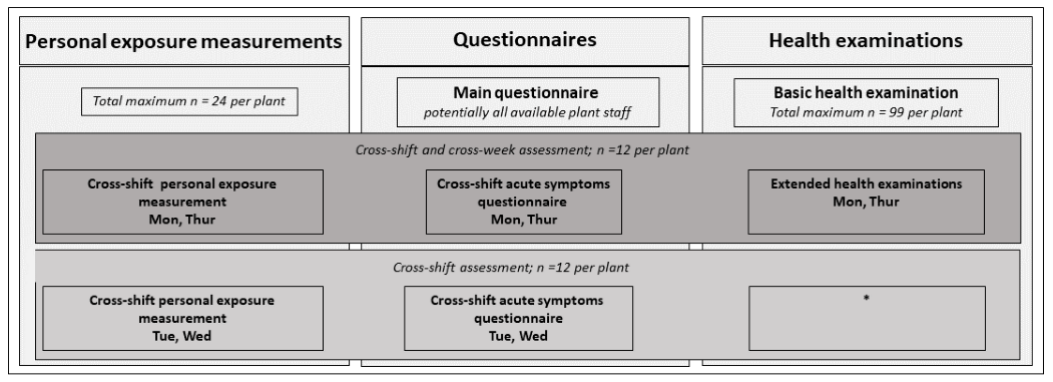
Overview of the different modes of data collection for both baseline and follow-up visits. Main questionnaires are to be filled out by all available plant staff, whereas exposure measurements and health examinations are restricted in number. All individuals carrying out cross-week exposure measurements on Monday and Thursday also undergo basic health examinations. *These staff carry out cross-shift exposure measurements on Tuesday and Wednesday; they can or cannot undergo basic health examinations.

The basic health examinations include skin prick test, spirometry, and blood samples. Workers participating in personal exposure measurements on Monday and Thursday undergo extended health examinations with cross-shift and cross-week assessments that include repeated spirometry, extended and repeated blood samples, and short questionnaires regarding acute symptoms (see Figure 2). Workers participating in personal sampling on Tuesday and/or Wednesday can or cannot undergo basic health examinations, depending on their preference. Health assessments are described in more detail in the following sections.

##### Lung Function

Spirometry, including forced expiratory volume in 1 second (FEV_1_) and forced vital capacity (FVC), is performed as recommended in the American Thoracic Society and European Respiratory Society guidelines [31]. In one of the geographical areas, it is intended to perform a reversibility test in a subsample of participants with abnormal lung function at T_2_. In addition, subsamples of about 80 participants with work-related lower respiratory symptoms and 20 participants with no respiratory symptoms from 6 plants are asked to perform serial peak expiratory flow (PEF) measurements for 4 weeks at least 4 times a day. The PEF data will be interpreted with the Occupational Asthma System (OASYS) software [32].

##### Respiratory Symptoms

Information on symptoms from the lower and upper airways in general, during the past week, or during the past 12 months are collected through the questionnaires comprising standardized questions on respiratory symptoms, allergic status, and malaise from the European Community Respiratory Health Survey (ECRHS). Participants wearing bioaerosol sampling equipment also answer questionnaires on acute symptoms from eyes, upper and lower airways modified from Wasserfallen et al [33] before and immediately after ending the work shift [34].

##### Hand Eczema and Urticaria

Symptoms of hand eczema and urticaria are assessed through the Nordic Occupational Skin Questionnaire (NOSQ-2002). In 1 of the 3 geographical areas, scoring of hand eczema using the Hand Eczema Severity Index (HECSI) [35] is carried out, and skin barrier function (degree of hydration) is assessed measuring transepidermal water loss (TEWL) [36].

##### Immunological and Inflammatory Markers

Blood samples are collected for determination of high-sensitive c-reactive protein (CRP), total IgE, as well as specific IgE in a pooled screening test of common inhalant allergens (1 for birch, timothy grass, mugwort, alternaria, and cladosporium, and 1 for cat, horse, dog, mites, and rabbit), as well as IgE against salmon, cod, *Anisakis simplex*, and shrimp. As part of the extended health examinations, peripheral blood leukocytes (total and differential count of lymphocytes, eosinophils, and neutrophils), and CRP are assessed cross-shift and cross-week (Monday and Thursday).

Skin prick tests are performed, including commercial tests for seafood components as well as in-house study-specific extracts from salmon components, to map immunological responses to common seafood components that workers are exposed to. The 10 substances tested comprise commercial cod and salmon with its positive and negative controls, as well as study-specific extracts made in the Tromsø laboratory (2 negative controls, raw muscle, mucous from skin, skin, and cooked muscle).

Figure 2 summarizes the different modes of data collection during fieldwork. For questionnaires and registration cards, see Multimedia Appendix 1. More details on different analytical methods and health assessments will be described in detail in future publications regarding the specific topics.

### Specifics for the Intervention Trial

The core of the study is a pragmatic parallel group, 3-arm nonrandomized multicenter superiority trial with a 1:1:1 allocation ratio (ClinicalTrials.gov NCT05039229).

#### Eligibility and Exclusion Criteria for Individual Participants

All workers employed in, and present at, the processing plants at baseline (T_1_) and follow-up (T_2_) are eligible for answering the self-administered questionnaire. The numbers of invitees for health examinations and individual exposure assessments are limited to a maximum of 99 and 24, respectively, per plant, owing to logistical, financial, and time constraints.

Pregnant participants are excluded from skin prick testing and spirometry, while persons with a history of heart attack or surgical interventions in the eye, stomach, or chest within the preceding 3 months are excluded from carrying out spirometry testing.

#### Intervention Groups and Content of Intervention Follow-Up

##### Overview

While the control group will not receive any intervention, there are 2 different intervention arms that imply measures with the potential to reduce bioaerosol exposure for the employees. These intervention arms represent a general intervention category in which details for the actual intervention are to be developed in collaboration with the factory management and staff. Their involvement ensures local knowledge, including local-specific characteristics, specific location of intervention, other ongoing projects, operation plans, or other aspects that are important to achieve a high effect of intervention measures.

The following sections describe possible alternatives for interventions.

##### Nozzles Intervention

Nozzles intervention (NZ) is a technical intervention targeting alteration of nozzle types, numbers, or nozzle function along the production line, for example, by manipulation of nozzle dimensions, alteration of operating pressure, or shielding of the nozzle’s output stream.

##### Cleaning of Surfaces Intervention

Cleaning of surfaces intervention (CS) is a behavioral or technical intervention related to cleaning of the workers’ personal operating areas (work benches and part of production lines) during work operations or while cleaning floor areas during production hours. This can include increased use of swabbing instead of flushing with water hoses or alterations in use of water hoses for cleaning. Examples for the latter are a reduced flushing frequency, reduction of number of employees executing the work, alterations of hose dimension, or nozzles on hoses.

##### Control Group

For the control group work is to be carried out as usual without any intervention measures.

Interventions in the NZ and CS groups were to be started between 5 and 8 weeks after baseline measurements at time point T_1_. The weeks between T_1_ and T_2_ are hereafter called “intervention weeks.” All 3 arms are followed up in the same way between T_1_ and T_2_. This includes regular contact by phone or email and 1 physical meeting in intervention weeks 31-36. This midway meeting has a scheduled agenda addressing evaluation of baseline fieldwork, suggestions and input for follow-up fieldwork, status of potential technical changes in the plant, as well as assessment of adherence and employees’ experiences of the intervention and study participation.

#### Adherence to the Trial Protocol and Concomitant Activities

Regular contact, midway meetings, and postintervention measurements are one way of increasing adherence in the intervention groups. Local staff involvement in the eventual design of the intervention also has the potential to increase adherence, especially when behavioral changes are part of the intervention.

Although it was expressed to the plants’ management that it is desirable to not engage in any other technical or behavioral changes that could affect the study, there are no restrictions regarding concomitant activities during the study period.

#### Recruitment of Processing Plants and Individual Participants

Participating plants were recruited through earlier collaboration and by establishing new contacts based on regional knowledge about existing factories.

Each plant has a designated contact person from their own staff who helps with recruitment of individual participants and organization and logistics related to fieldwork at T_1_ and T_2_; this person keeps in contact throughout the intervention period.

#### Timeline

Participating factories were recruited during time of funding application. A visit at time point 0 (T_0_), 1-3 weeks before baseline measurements at T_1_, was used for detailed mapping of the specific factory’s infrastructure, a walk-through of the facilities, and for recruiting individual participants through information meetings and distribution of information material. Allocation of intervention groups was done based on this mapping and communicated to the factories in a meeting with company, employee, and safety representatives during the week of baseline measurements at T_1_. The companies then had time to discuss details of the intervention activities internally until the expected implementation period (intervention weeks 5-8).

Individual participants are enrolled with individual consent forms for both health examinations and questionnaires. T_1_ and T_2_ measurements were scheduled within 1 working week (Monday through Thursday). The interval between T_1_ and T_2_ is set to be 12 months (plus or minus 2 weeks).

Table S1 in Multimedia Appendix 2 gives an overview of the timeline of the intervention study.

#### Assignment of Intervention

Randomization of intervention arms was not feasible because of different premises of plant infrastructure. The participating plants were therefore allocated to the study arms by the project group, based on meetings and discussion with the local staff and management, and aiming at covering all 3 study arms in each region. Criteria that were taken into account for allocation to a specific arm were prestudy status of awareness and compliance of bioaerosol-reducing behaviors and technical installation, such as use of swabbing; flushing of work areas during work shifts; number, type, and shielding of water nozzles; water pressure; ventilation system; and plans for any alteration of these factors within the study period.

Allocation concealment and blinding was not relevant for obvious reasons.

### Data Collection and Management

#### Database

Study data are collected and managed using REDCap electronic data capture tools hosted at the University Hospital of North Norway (UNN) [37,38]. REDCap is a secure, web-based software platform designed to support data capture for research studies; it provides (1) an intuitive interface for validated data capture; (2) audit trails for tracking data manipulation and export procedures; (3) automated export procedures for seamless data downloads to common statistical packages; and (4) procedures for data integration and interoperability with external sources. The main questionnaire and the questionnaires on acute symptoms are filled out in a self-administered paper form and consecutively scanned into the REDCap database. Analysis results on occupational exposure, inflammation markers (blood), skin examinations, skin prick tests, and spirometry will be put into the system by import or manual plotting.

We use the automatic quality check inherent in REDCap, including checking that critical variables have been entered, checking that the participant has consented, and performing range checks and inspection of outliers. Visual data checks are performed for 10% of the manually plotted data in order to identify transcription errors (skin prick tests and lung function measures). Exposure measurements, differential counts, CRP, and IgE are visually checked (100%) after transfer to Excel (Microsoft Corp) sheets. Excel sheets are imported into RedCap and 10% are checked after this process. In addition, 10% of the main questionnaires that are scanned and directly imported into REDCap will be visually inspected and adjudicated.

The database is administered at UNN and will comprise all the original data from T_1_ and T_2_, in addition to metadata and syntaxes (statistical coding) for generated variables. Only a limited number of researchers will have access to the data, as well as have permission to do data entries and corrections.

#### Biobank

Biological specimens (serum and plasma) are transferred and stored in a pseudonymized form in a project specific biobank at UNN for later analyses.

#### Data Management Group

The project has established a data management group comprising of the principal investigator and leaders of the 6 work packages (WPs). If not covered by the group of WP leaders, each center is represented by up to two other researchers. The data management group will ensure that the publication plan covers the research questions in the protocol, handle applications of data and biomaterial, and make sure that the data management in the project is in compliance with the approvals from the ethical board and General Data Protection Regulation (GDPR). A data management plan further describes data security issues and ethics regarding data management.

#### Ownership and Use of Data

As this project is a multicenter study, an agreement of ownership and use of data has been completed among the 3 study centers. Agreements between the 3 study centers and the participating plants, ensuring mutual commitment to its collaboration, have also been signed. All data will be archived for 10 years after study termination. Extension of data archives beyond 10 years must seek special approval by the Norwegian ethical committees.

#### Data Access

The participant-level data set will not be available for public access owing to the GDPR. Metadata and statistical codes (syntaxes) beyond those reported in publications will be available upon reasonable request.

### Ethics Approval

This research is carried out in compliance with the Helsinki Declaration of 1975 as revised in 2000, and has been approved by the Regional Committee for Medical Research Ethics North Norway (REK Nord No. 175081). Written, informed consent is obtained from each participant before participation. The consent forms emphasize the right to withdraw from the study at any time without explanation, and that the participants may at any time request that their biological material be destroyed. Information about the study was given orally in an information meeting at T_0_ and orally and in written form during fieldwork (T_1_ and T_2_), with the opportunity to ask questions on-site. Personally identifiable data are entered and analyzed in pseudonymized form by replacing the name with a project-specific identification code, ensuring personal confidentiality. Only the principal investigator and co–principal investigator are given access to a code key that is stored electronically with 2-factor authentication on a safe server at UNN. All personal data will be handled in accordance with GDPR.

The project protocol will ensure that findings reported back to the participants will be analytically valid, clinically significant, and actionable [39,40]. Abnormal lung function measurements, positive skin prick tests, and IgE for salmon-related proteins, and white blood cell count substantially outside the normal range, as well as abnormal white blood cell distribution will be reported back to the participants.

There are ethical concerns related to the common use of workers from outside Norway hired from temporary staff recruitment agencies, as workers with occupational health issues discovered in the project may be lost to follow-up. We will try to avoid this by giving appropriate information at a personal level. All data collection and result analyses will be performed or supervised by experienced investigators trained in good ethical research practices. Scientific and popular scientific publishing will follow the ethical standards for coauthorship and publishing.

### Statistical Analysis

#### Sample Size and Power Analysis

Statistical power was estimated according to the primary outcomes of the project: the change in exposure to inhalable total protein in the breathing zone before T_1_ and after T_2_ and implementing exposure-reducing interventions in the participating salmon processing factories. A previous study in the Norwegian salmon industry reported a geometric mean exposure of total protein in the processing areas of 2.7 (range 0.76-12.62) µg/m^3^ [4]. The study was based on 273 exposure measurements, making it the most extensive study available on assessment of the concentration of total proteins in the salmon processing workers’ breathing zone. The crude data allowed us to estimate the corresponding arithmetic mean exposure of total protein (10.0, SD 5.8 µg/m^3^). Based on these data we estimated that with a significance level of .05 there is a power of 80% of finding a 40%, 30%, and 20% reduction of exposure in 1 intervention arm if we include 34, 59, and 133 measurements in each of the groups, respectively. Since baseline measurements are performed at all sites before intervention, there will be an even higher statistical power given that we can adjust the exposure levels after intervention for mean baseline values at each site. Based on these calculations, we aimed at including 24 workers with 2 repeated measurements in each of the intervention groups (144 measurements in each group equals a total of 432 measurements) both at baseline and after intervention.

#### Analysis of Data

Detailed description of statistical analyses will be found in the respective scientific publications. In general, for parametric data displaying a normal distribution, results will be presented as arithmetic means with SDs. For data showing a skewed distribution, as is commonly found for exposure measurement data, the results will be presented as geometric means with geometric SDs. Categorical data will be presented as percentages. In case of missing variables, omission or imputation will be performed depending on the type of data.

In general, risk estimates for the various outcomes with 95% CIs will be estimated using a regression model being appropriate for the analysis in question. The estimates will be adjusted for covariates identified as potential confounders, effect modifiers, and mediators. Tests are considered statistically significant at a *P* value of .05.

Analyses related to the intervention study will be performed according to intention-to-treat, regardless of protocol adherence. Results will be reported in line with CONSORT (Consolidated Standards of Reporting Trials) guidelines.

#### Intervention Outcomes

The primary outcome, outcome measure (OM) 1, is to evaluate the effect of the interventions (control measures) on reducing salmon processing workers’ personal exposure to airborne inhalable total protein (µg/m^3^) from T_1_ to T_2_. Differences in mean concentration of full shift measurements (8 hours) between intervention groups and between intervention groups and control groups will be analyzed.

The secondary outcome measures are the changes from T_1_ to T_2_ for a range of other exposure and health outcomes. Differences between intervention groups and between intervention groups and control groups will be analyzed. The following secondary outcomes will be assessed:

OM 2: Change of concentration of salmon processing workers’ personal exposure to proteases (ng/m^3^).

OM 3: Change in total protein (µg/m^3^, inhalable aerosol fraction) from stationary measurements in relevant areas of the salmon production line.

OM 4: Change of salmon processing workers’ personal exposure to airborne fish allergens, measured by concentration of total aerosol fraction of fish allergens (ng/m^3^).

OM 5: Change of salmon processing workers’ personal exposure to airborne endotoxins (EU/m^3^).

OM 6: Change in salmon processing workers’ self-reported symptoms from the upper airways.

OM 7: Change in salmon processing workers’ self-reported symptoms from the lower airways.

OM 8: Change in salmon processing workers’ self-reported eye symptoms.

OM 9: Change in salmon processing workers’ lung function at group level (FEV_1_ in percentage of predicted value).

OM 10: Change in salmon processing workers’ lung function at individual level (FEV_1_/FVC ratio).

OM 11: Change in salmon processing workers’ cross-shift lung function (FEV_1_).

OM 12: Change in salmon processing workers’ cross-week lung function (FEV_1_).

OM 13: Incidence of salmon processing workers’ sensitization to salmon assessed by serum IgE specific to salmon proteins.

OM 14: Change in salmon processing workers’ self-reported skin symptoms.

For more details regarding the intervention study, please see ClinicalTrials.gov NCT05039229.

### Other Outcomes of Interest (Substudies)

In addition to outcome measures related to the intervention study itself, substudies will investigate the following:

1. Characterization of airborne bioaerosols from the work environment with respect to inhalable protein, protease enzymes, microorganisms, and microbially derived agents, as well as known and possible novel fish allergens.
2. Development of improved test extracts for seafood allergy diagnostics
3. Exposure-response relationship between bioaerosol exposure and indicators of respiratory health effects, markers of inflammation, and sensitization.
4. Identification of factors that can explain the variation in bioaerosol exposure (determinants of exposure).
5. Evaluation of prevalence and determinants of respiratory work-related symptoms and disease.
6. Evaluation of prevalence of work-related asthma by assessment of respiratory symptoms and work-related airflow limitation by serial measurements of PEF.
7. Cross-shift and cross-week changes in acute symptoms from airways and eyes, as well as inflammatory biomarkers.
8. Status of markers of inflammation and sensitization among salmon processing workers.
9. Presence of indicators of health-promoting working environments in the salmon processing industry, demographical differences in perception, and their consistency through a 1-year follow-up.
10. Evaluation of prevalence and determinants of dermatological work-related symptoms and disease.
11. Objective evaluation of hand eczema and skin barrier function.
12. Investigation of which indicators of health-promoting working environments are present in the salmon processing industry, and whether these are consistent through a 1-year follow-up.
13. Associations between employment conditions and health among migrant workers.

### Participant and Public Involvement

Participants and the public have been involved in the design of and recruitment for the study by personal contact and through the reference group. They will be further involved in dissemination activities to ensure that information is given in a way that is clear and easy to understand for the different stakeholders.

### Reference Group

The reference group’s mandate is to ensure that the project is relevant for the salmon processing workers, is professionally sound and anchored within the industry, and that it is practically feasible and adequately prioritized. Its main role is to give advice in terms of planning, executing, and possibly adjusting the project. The group’s purpose it to ensure good collaboration with those who can profit from the project’s results, to root the project in the participating factories, as well as to give advice in terms of communication. The reference group does not have any responsibility for the project’s progression, goal achievement, or formalities; neither does it have any authority to take decisions. It consists of members of the employer organization the Norwegian Seafood Federations (Sjømat Norge), the employee organization Norwegian Food and Allied Workers Union (Norsk Nærings- og nytelsesmiddelarbeiderforbund), the Norwegian Labour Inspection Authority, an occupational health service, 1 plant representative from each of the 3 geographical regions, 1 researcher from each of the project group’s locations (Tromsø, Bergen, Trondheim), and the principal investigator.

## Results

The study was originally planned to begin in fall 2020 but was delayed to September 2021 due to the COVID-19 pandemic. The pandemic further influenced both data gathering and company access due to access restrictions and short-notice sick leaves or quarantines. Among other consequences, this led to delays in baseline measurements, especially in the plants in mid-Norway that had carried out their baseline measurements (T_1_) between March and June 2022, 3 months behind schedule.

As the study is still ongoing and data quality checking is not finished, we only have preliminary results from the baseline measurements at T_1_. The main questionnaire at T_1_ was answered by 740 participants. Due to uncertainties in numbers of distributed questionnaires, it was not possible to calculate a precise response rate, but we estimated its average to be 61% with a range between about 48 (31%) out of 155 and 134 (91%) out of 147. A total of 673 workers participated in the baseline health examinations. Calculating the participation rate is futile, because in several plants there was a much higher number of potential participants than there was capacity to carry out health examinations.

Exposure measurements were undertaken, resulting in over 700 personal measurements (400, 203, and 122 for protein, allergens, and endotoxins, respectively), as well as almost 150 stationary measurements (59, 51, and 34 for protein, allergens, and endotoxins, respectively).

Due to a long time frame of about 2 years between the companies’ original consent to participate in the trial and the actual start of the study, technical adjustments were done in several factories before the study start. Thus, the original plan to allocate 3 plants per intervention group was not feasible. One plant planned adjustments to the ventilation system during the intervention period and was therefore not allocated to any of the original intervention groups at T_0_. This left only 2 groups for the CS arm, while 3 plants each were allocated to the NZ and control group arms.

## Discussion

### Preliminary Principal Findings

The response rate could not be calculated precisely as the project group did not have the opportunity to distribute questionnaires personally in all plants. Also, the seafood industry is an industry heavily relying on short-time and temporary labor, making it difficult to keep track of which employees are available for inclusion at any given time, as well as to supply questionnaires in the correct language. Difficulties in calculating exact response and participation rates is a known problem in research concerning occupational health in the seafood industry [41,42], and our average is in line with earlier studies with questionnaires in the Norwegian seafood industry [43].

In general, response or participation rates have been declining steeply over the past decades. It has been suggested to report several types of rates together with careful explanations about how they were calculated, rather than reporting single numbers, in order to give a more nuanced picture of the specific study [44].

### Strengths and Limitations

To our knowledge, this is the largest study on a salmon processing worker population assessing exposure to bioaerosols and its associations with respiratory and skin outcomes. Further, it is the first study to strategically test different measures of intervention for reducing bioaerosol levels.

The main strengths of the study include (1) its prospective design, with measurements of bioaerosol exposure and a range of health effects before and after implementation of exposure-reducing measures; (2) its multicenter design and provision of study material in 6 relevant languages, thereby covering a large and representative proportion of workers in the Norwegian salmon processing industry; (3) the large population size, with repeated sampling over consecutive work shifts and with a 1-year interval between measurements, which allows us to assess what affects the variability in exposure; and (4) extensive collection and analysis of active components in the bioaerosols, such as fish allergens, enzymes, and endotoxins, which will allow us to investigate which bioactive molecules and microbial agents are commonly present in bioaerosols produced during salmon processing, as well as respiratory and dermal health and health-promoting factors among salmon industry workers. The repeated measurements design allows analyses of paired samples, reducing the interindividual variation in samples assumed to show small changes in assumed healthy workers.

A special trait of the study is the combination of the assessment of work-related risk factors and work-related health-promoting factors which has so far not been done in the salmon processing industry.

For the health examinations, we cannot rule out a certain selection bias, as participation was restricted due to capacity reasons, as well as because it was voluntary. The latter could result in workers with existing health problems being more prone to participate, in order to find out more about their health, but it could also lead to refusal due to a fear of losing their job. The latter was addressed in the consent form and information meetings, emphasizing that employers of plant staff will not be informed about individual health findings. In any case, it is impossible to say in which direction this possible bias would influence results.

The lack of randomization to interventional categories is in part a weakness, but at the same time a necessary approach in order to make the study feasible. The seafood industry is in constant change and has to react quickly to challenges on the market. Therefore, it is unlikely to meet stable conditions over a longer time period, as this study confirmed. Also, as each plant’s infrastructure and needs are so different, it is not feasible to allocate the plants to predefined detailed interventions. Due to limited capacity, it will only be possible to include some of the participants in the different health examinations, and this will hamper the interpretation of associations between the different outcomes to a varying extent.

Another weakness is the pandemic-related delay in baseline measurements at 3 of the plants, which moved the period under study forward to a timeframe with a potentially lower production rate, warmer weather, and higher probability of pollen exposure outdoors, which could affect exposure measurements, as well as symptoms in the airways and skin. In order to keep circumstances somewhat similar, follow-up measurements at T_2_ are planned to be performed around the same time of the year.

All abovementioned factors will be further discussed in future publications for data from this study.

### Dissemination Plan

Trial results will be disseminated to participants, the salmon processing industry, researchers, health personnel, authorities, and other interested parties through scientific conferences, publications, reports, and public dissemination measures. There are no publication restrictions, and the results will be disseminated regardless of the magnitude or direction of the effect. Authorship eligibility is determined according to the International Committee of Medical Journal Editors criteria for manuscripts submitted for publication. There is no intention to use professional writers. Data presentation will be performed in a way that ensures confidentiality for individual or workplace-specific data.

## Appendix

Multimedia Appendix 1 Main questionnaire, pre- and postshift questionnaire.

Multimedia Appendix 2 Timeline of participants and data gathering.

Multimedia Appendix 3 Peer-review reports from the Research Council of Norway (Norway).

## Abbreviations

BCA: bicinchoninic acid
CONSORT: Consolidated Standards of Reporting Trials
CRP: c-reactive protein
CS: cleaning of surfaces intervention
EAACI: European Academy of Allergy and Clinical Immunology
ECRHS: European Community Respiratory Health Survey
EU: endotoxin units
FEV_1_: forced expiratory volume in 1 second
FVC: forced vital capacity
GDPR: General Data Protection Regulation
HECSI: Hand Eczema Severity Index
IFISH5: Fifth International Fishing Industry Safety and Health Conference
IgE: immune globulin E
LC-MS: liquid chromatography–mass spectrometry
MALDI-TOF MS: matrix-assisted laser desorption and ionization-time of flight mass spectrometry
NOSQ-2002: Nordic Occupational Skin Questionnaire
NZ: nozzles intervention
OASYS: Occupational Asthma System
OM: outcome measure
PEF: peak expiratory flow
rFC: recombinant factor C
SDS-PAGE: sodium dodecyl-sulfate polyacrylamide gel electrophoresis
T1: time point 1 of intervention trial (baseline)
T2: time point 2 of intervention trial (follow-up)
TEWL: transepidermal water loss
UNN: University Hospital of North Norway
UWES: Utrecht Work Engagement Scale
WEMS: Work Experience Measurement Scale
Work-SOC: Work-related Sense of Coherence Scale
WP: work package
