# Supplementary material for: Effects of Interventions to Prevent Work-Related Asthma, Allergy, and Other Hypersensitivity Reactions in Norwegian Salmon Industry Workers (SHInE): Protocol for a Pragmatic Allocated Intervention Trial and Related Substudies
Source: JMIR Res Protoc. 2023 Jul 19;12:e48790. doi: 10.2196/48790 (PMC10398556; doi:10.2196/48790)
Supplement: Multimedia Appendix 1 [file resprot_v12i1e48790_app1.pdf]

We used the same questionnaire at your workplace about 1 year ago.

Did you answer the questionnaire 1 year ago? ☐ **NO** ☐ **YES**

(Please complete the entire questionnaire regardless of what you did last time).

Personal details and general information

1. What is your date of birth? (day/month/year) ☐☐ / ☐☐ / ☐☐☐☐
   dd mm yyyy

2. What is today's date? (day/month/year) ☐☐ / ☐☐ / ☐☐☐☐
   dd mm yyyy

3. Have you been at work during the past week? ☐ NO ☐ YES

4. Gender: ☐ Man ☐ Woman

5. How tall are you? ☐☐☐ cm

6. How much do you weigh? ☐☐☐ kg

7. Which country are you from?

   ☐ Norway

   ☐ Poland

   ☐ Lithuania

   ☐ Latvia

   ☐ Slovakia

   ☐ Romania

   ☐ Bulgaria

   ☐ Other country

8. Language while at work

   Circle the **number** that best describes your feelings.

   A. How much is language a barrier for you when you are at work?

   Not very much 1 2 3 4 5 6 A lot

   B. How much is language a barrier to communication with the management?

   Not very much 1 2 3 4 5 6 A lot

   C. How good is your command of the language you use when at work?

   Not very good 1 2 3 4 5 6 Very good

9. What is your highest level of education?
   (mark one alternative only)

☐ Primary and lower secondary school

☐ Upper secondary school, vocational school, technical college or equivalent

☐ University college or university

Occupation, working conditions and exposure

CURRENT JOB

10. When did you start at your current workplace? ☐☐ mm ☐☐☐☐ yyyy

11. What type of employment contract do you have?

☐ Zero hours contract

☐ Permanent contract/position

☐ Other

12. Do you have a managerial position?

☐ NO ☐ YES

A. If YES:

☐ Manager

☐ Middle manager

13. How many hours per week do you work in your job (on average)? ☐☐,☐ hours/week

14. In which department do you work?
   (Select as many alternatives as apply)

☐ Slaughterhouse

☐ Filleting

☐ Other processing besides filleting

☐ Cleaning

☐ Packing/freezer room/warehouse

☐ Maintenance of machinery/production equipment

☐ Administration/office

☐ Other

15. What duties do you perform and/or which machines/processes do you supervise in your current job?
(Mark one alternative in each row; answer as precisely as you can)

	Daily	Weekly (at least once a week, but not every day)	Every other week or less often/never
Tend to live salmon (røkting)	☐	☐	☐
Bleeding	☐	☐	☐
Gutting (on gutting machine or manually)	☐	☐	☐
Manual cleaning after gutting	☐	☐	☐
Decapitation	☐	☐	☐
Descaling/scale off (removal of fish scales)	☐	☐	☐
Desliming (removal of fish slime)	☐	☐	☐
Cutting, cutting of fillets using machinery	☐	☐	☐
Use of machinery based on water jet technology	☐	☐	☐
Fillet cutting/trimming by hand	☐	☐	☐
Sorting by weight/quality control	☐	☐	☐
Packing of whole/fresh fish	☐	☐	☐
Work in cold rooms/freezer rooms/ warehouses	☐	☐	☐
Handling of fish waste	☐	☐	☐
Technical maintenance of machinery/production equipment	☐	☐	☐
Technical process management	☐	☐	☐
Operating forklift/ "snile" indoors	☐	☐	☐
Operating forklift outdoors (electric or diesel)	☐	☐	☐
Hosing floors, storage containers, production lines	☐	☐	☐
Hosing down worktables	☐	☐	☐
Cleaning of production premises	☐	☐	☐
Cleaning of other company facilities: (e.g. offices, meeting rooms, changing rooms, canteen)	☐	☐	☐
Laboratory work	☐	☐	☐
Office/administrative tasks	☐	☐	☐
Canteen work	☐	☐	☐
Process control in production areas (linjeleder)	☐	☐	☐

16. Do you currently wear gloves and/or cuffs for work?
(Mark one alternative in each row)

	Hours per day (on average)		
	0 hours (Don't wear them)	Less than 2 hours	2 hours or more
Disposable gloves	☐	☐	☐
Thick work gloves	☐	☐	☐
Cotton gloves as inner gloves	☐	☐	☐
Cuffs/forearm protection	☐	☐	☐

17. What do you currently come into contact with when not wearing gloves while you are at work?
(Mark one alternative in each row)

	Hours per day (on average)		
	0 hours	Less than 2 hours	2 hours or more
Water	☐	☐	☐
Salmon flesh	☐	☐	☐
Salmon slime	☐	☐	☐
Salmon blood/entrails	☐	☐	☐
Detergents, cleaning agents or disinfectants for industrial use	☐	☐	☐
Oils (in connection with technical maintenance work)	☐	☐	☐

18. How many times do you wash your hands during a usual working day?
(include hand washing during your work and at home/outside work)

	At work	At home/outside of work
0-5 times per day	☐	☐
6-10 times per day	☐	☐
11-20 times per day	☐	☐
More than 20 times per day	☐	☐

19. How often do you use disinfectant/hand sanitizer during a typical working day?
(Include use of disinfectant/hand sanitizer during your work and at home/outside work)

	At work	At home/outside of work
0-5 times a day	☐	☐
6-10 times a day	☐	☐
11-20 times a day	☐	☐
More than 20 times a day	☐	☐

PREVIOUS JOB

20. Have you worked in the seafood industry before? ☐ NO ☐ YES

A. If YES, in what part of the seafood industry?

☐ Salmon

☐ Whitefish

☐ Shellfish

☐ Other

21. How many years have you worked with salmon (both now and in the past)

☐☐ year ☐☐ months

Smoking habits

22. Do you smoke? (this applies even if you only smoke the odd cigarette every week) ☐ NO ☐ YES

23. Did you smoke previously? ☐ NO ☐ YES

If YES to question 22 and/or question 23:

A. How much do or did you smoke? (give an average) ☐☐ cigarettes/day

(*give an average*)

B. How old were you when you started smoking? ☐☐ years

C. For how long have you smoked? (applies to both smokers and ex-smokers) ☐☐ years

D. If you are an ex- smoker, how old were you when you stopped smoking? ☐☐ years

51375

Respiratory and allergic symptoms

24. Have you had wheezing or whistling in your chest at any time **in the last 12 months**?

☐ NO ☐ YES

If YES:

A. Have you been at all breathless when the wheezing noise was present?

☐ NO ☐ YES

B. Have you had this wheezing or whistling when you did not have a cold?

☐ NO ☐ YES

25. Have you had an attack of shortness of breath that came on **following** strenuous activity at any time in the last **12 months**?

☐ NO ☐ YES

26. Have you woken up with a feeling of tightness in your chest at any time **in the last 12 months**?

☐ NO ☐ YES

27. Have you been woken by an attack of shortness of breath at any time **in the last 12 months**?

☐ NO ☐ YES

28. Have you been woken by an attack of coughing at any time **in the last 12 months**?

☐ NO ☐ YES

29. Do you have or have you ever had asthma?

☐ NO ☐ YES

If YES:

A. Have you ever had asthma diagnosed by a doctor? ☐ NO ☐ YES

B. How old were you when you first experienced asthma symptoms? ☐☐ years

C. How old were you when you last experienced asthma symptoms? ☐☐ years

30. Have you had an attack of asthma **in the last 12 months**?

☐ NO ☐ YES

31. Are you currently taking any medicine (including inhalers, aerosols or tablets) for asthma?

☐ NO ☐ YES

32. When you are at work, do you ever

A. Start to cough?

☐ NO ☐ YES

B. Start to wheeze?

☐ NO ☐ YES

C. Start to feel short of breath or get chest tightness?

☐ NO ☐ YES

D. Do you sometimes experience any of the above symptoms (cough, wheezing in the chest, difficulty breathing or tightness of the chest) in the afternoon, evening or night after you have been to work?

☐ NO ☐ YES

If "YES" to one of the statements A-D:

E. Do these problems related to your work lessen or disappear during the weekend or during holidays?

☐ NO ☐ YES

33. Have you ever experienced nasal symptoms such as nasal congestion, rhinorrhoea (runny nose) and/or sneezing attacks without having a cold?

☐ NO ☐ YES

If YES:

A. How old were you when you experienced such nasal symptoms for the first time? ☐☐ years

B. Have you had such nasal symptoms **in the last 12 months**?

☐ NO ☐ YES

C. Do these symptoms decrease or disappear over a weekend or while you are on holiday?

☐ NO ☐ YES

D. Has this nose problem been accompanied by itchy or watery eyes?

☐ NO ☐ YES

E. At what time of year are your nose symptoms worst?

☐ Spring

☐ Summer

☐ Autumn

☐ Winter

☐ All year round

☐ Don't know

34. Do you have any nasal allergies including hay fever?

☐ NO ☐ YES

35. Have you had flu-like symptoms at work or after work during **the last 12** months, even though you did not have a cold or any other infectious disease?
(Flu-like symptoms include fever, chills, nausea, muscle/joint pain)

☐ NO ☐ YES

If YES:

A. Did these symptoms decrease or disappear over a weekend or while you were on holiday?

☐ NO ☐ YES

36. During **the past week**, have you experienced any of the following symptoms **during your working day**?

Grade the symptoms (circle the appropriate number) on a scale from 0 (no symptoms) to 4 (severe symptoms):

Eye symptoms

A. Itching

No symptoms 0 1 2 3 4 Severe symptoms

B. Swelling

No symptoms 0 1 2 3 4 Severe symptoms

C. Discharge

No symptoms 0 1 2 3 4 Severe symptoms

Nose symptoms

D. Itching

No symptoms 0 1 2 3 4 Severe symptoms

E. Sensation of fullness, congestion, or blockage

No symptoms 0 1 2 3 4 Severe symptoms

F. Sneezing

No symptoms 0 1 2 3 4 Severe symptoms

G. Discharge or runny nose

No symptoms 0 1 2 3 4 Severe symptoms

Respiratory symptoms

H. Cough

No symptoms 0 1 2 3 4 Severe symptoms

I. Wheeze (whistling in your chest)

No symptoms 0 1 2 3 4 Severe symptoms

J. Mucus which is difficult to cough up

No symptoms 0 1 2 3 4 Severe symptoms

K. Shortness of breath or chest tightness

No symptoms 0 1 2 3 4 Severe symptoms

37. Did you suffer from a cold in the last week?

☐ NO ☐ YES

38. Have you ever been ill or experienced symptoms within two hours of eating salmon? (e.g. rash, itching, diarrhoea, vomiting, runny or stuffy nose, wheezing)

☐ NO ☐ YES

If YES:

A. Have you almost always been ill or experienced these symptoms within two hours of eating salmon?

☐ NO ☐ YES

Skin symptoms

39. Do you have dry skin?

☐ NO ☐ YES

40. Have you ever had an itchy rash that has been coming and going for at least 6 months, and at some time has affected skin creases?
(by skin creases we mean folds of elbows, behind the knees, fronts of ankles, under buttocks, around the neck, ears, or eyes)

☐ NO ☐ YES ☐ DON'T KNOW

41. Have you had any of the following symptoms on your hands or wrist/forearms during **the past 12 months**?
(mark in each column any that are applicable)

	Hands	Wrist/forearms
No symptoms during the past 12 months	☐	☐
Redness	☐	☐
Dry skin with scaling/flaking	☐	☐
Fissures or cracks	☐	☐
Weeping or crusts	☐	☐
Tiny water blisters (vesicles)	☐	☐
Papules	☐	☐
Rapidly appearing itchy wheals/welts (urticaria)	☐	☐
Itching	☐	☐
Burning, prickling, or stinging	☐	☐
Tenderness	☐	☐
Aching or pain	☐	☐

42. Did you have eczema as a child? (also known as atopic eczema)

☐ NO ☐ YES ☐ DON'T KNOW

43. Have you ever had <u>hand eczema</u>?

☐ NO ☐ YES

44. Have you ever had <u>eczema on your wrists or forearms</u> (excluding fronts of elbows)?

☐ NO ☐ YES

If YES to question 43 and/or question 44:

How do you grade your eczema on a scale from 0 (no eczema) to 10 (extremely bad eczema)?
Circle the appropriate number.

A. Today:

No eczema 0 1 2 3 4 5 6 7 8 9 10 Extremely bad eczema

B. At worst:

No eczema 0 1 2 3 4 5 6 7 8 9 10 Extremely bad eczema

C. Does your eczema improve when you are away from your normal work (for example weekends or longer periods)?
(One answer in each column if applicable)

	Hand eczema	Wrists/Forearm eczema
NO	☐	☐
YES, sometimes	☐	☐
YES, usually	☐	☐
DON'T KNOW	☐	☐

45. Have you ever had **itchy wheals (urticaria)** appearing and disappearing rapidly (within hours) on your hands, wrists or forearms (urticaria or nettle rash)

☐ NO ☐ YES

If YES,

A. Have **itchy wheals (urticaria)** appeared on your hands, wrists or forearms after your skin has come into contact with any of the following at work?
(Mark in each column any that are applicable)

	NO	YES	DON'T KNOW
Salmon	☐	☐	☐
Disposable gloves/work gloves	☐	☐	☐
Hand disinfection	☐	☐	☐
Detergents, cleaning agents or disinfectants for industrial use	☐	☐	☐

46. During **the past week**, have you experienced itchy wheals (urticaria) on your hands, wrists or forearms **during your working day**?

Grade the symptoms (circle the appropriate number) on a scale from 0 (no symptoms) to 4 (severe symptoms):

No symptoms 0 1 2 3 4 Severe symptoms

General health

47. How would you assess your general health compared with other people your own age?

☐ Much better

☐ A little better

☐ About the same

☐ Slightly worse

☐ Much worse

48. Have you ever tested positive for COVID-19?

☐ NO ☐ YES ☐ DON'T KNOW

Well-being at work

49. How do you personally find your current job and work situation in general?
Circle the **number** that best describes your feelings.

A.	Unmanageable	1	2	3	4	5	6	7	Manageable
B.	Meaningless	1	2	3	4	5	6	7	Meaningful
C.	Unstructured	1	2	3	4	5	6	7	Structured
D.	Impossible to influence	1	2	3	4	5	6	7	Easy to influence
E.	Insignificant	1	2	3	4	5	6	7	Significant
F.	Unclear	1	2	3	4	5	6	7	Clear
G.	Uncontrollable	1	2	3	4	5	6	7	Controllable
H.	Unrewarding	1	2	3	4	5	6	7	Rewarding
I.	Unpredictable	1	2	3	4	5	6	7	Predictable

50. The following 9 statements are about how you feel at work. Please read each statement carefully and decide if you ever feel this way about your job. If you have never had this feeling, circle the "0" (zero) in the space after the statement. If you have had this feeling, indicate how often you feel it by circling the number (from 1 to 6) that best describes how frequently you feel that way.

0= Never
1= Almost never/ A few times a year or less
2= Rarely/ Once a month or less
3= Sometimes/ A few times a month
4= Often/ Once a week
5= Very often/ A few times a week
6= Always/ Every day

A. At my work, I feel bursting with energy

0 1 2 3 4 5 6

B. At my job, I feel strong and vigorous

0 1 2 3 4 5 6

C. I am enthusiastic about my job

0 1 2 3 4 5 6

D. My job inspires me

0 1 2 3 4 5 6

E. When I get up in the morning, I feel like going to work

0 1 2 3 4 5 6

F. I feel happy when I am working intensely

0 1 2 3 4 5 6

G. I am proud of the work that I do

0 1 2 3 4 5 6

H. I am immersed in my work

0 1 2 3 4 5 6

I. I get carried away when I'm working

0 1 2 3 4 5 6

51. Supportive Working Conditions
Please enter your opinion on the following statements about your work situation.
The further to the right you put your response tick, the more you agree with the statement.
Circle the appropriate number.

A. We encourage and support each other at work

Totally disagree 1 2 3 4 5 6 Totally agree

B. There is a good atmosphere where I work

Totally disagree 1 2 3 4 5 6 Totally agree

C. I think the work routines function well

Totally disagree 1 2 3 4 5 6 Totally agree

D. I get feedback on the work I do

Totally disagree 1 2 3 4 5 6 Totally agree

E. I am happy about my job

Totally disagree 1 2 3 4 5 6 Totally agree

F. I feel that my employer invests in my health

Totally disagree 1 2 3 4 5 6 Totally agree

G. I get advice and practical help from others when necessary

Totally disagree 1 2 3 4 5 6 Totally agree

52. Internal Work Experience
Please enter your opinion on the following statements about your work situation.
The further to the right you put your response tick, the more you agree with the statement.
Circle the appropriate number.

A. I feel that my work is meaningful

Totally disagree 1 2 3 4 5 6 Totally agree

B. I feel that my work situation makes me grow

Totally disagree 1 2 3 4 5 6 Totally agree

C. There is variety in my work

Totally disagree 1 2 3 4 5 6 Totally agree

D. I do the work I was trained for

Totally disagree 1 2 3 4 5 6 Totally agree

E. I am happy when I go to work

Totally disagree 1 2 3 4 5 6 Totally agree

F. My work is a great personal challenge

Totally disagree 1 2 3 4 5 6 Totally agree

53. Autonomy
Please enter your opinion on the following statements about your work situation.
(Supply your entries based on your position or your work task.)
The further to the right you put your response tick, the more you agree with the statement.
Circle the appropriate number.

A. I decide **when** to do the various work tasks

Totally disagree 1 2 3 4 5 6 Totally agree

B. I decide **what** to do in my work

Totally disagree 1 2 3 4 5 6 Totally agree

C. I decide **how** to do my work

Totally disagree 1 2 3 4 5 6 Totally agree

D. I decide my own work pace

Totally disagree 1 2 3 4 5 6 Totally agree

54. Time Experience
Please enter your opinion on the following statements about your work situation.
The further to the right you put your response tick, the more you agree with the statement.
Circle the appropriate number.

A. I have enough time during my normal working hours to do my job without time pressure (stress)

Totally disagree 1 2 3 4 5 6 Totally agree

B. I always have time to finish each work task in the way it is supposed to be done

Totally disagree 1 2 3 4 5 6 Totally agree

C. I do not need to work more than my scheduled hours

Totally disagree 1 2 3 4 5 6 Totally agree

55. Leadership
Please enter your opinion on the statements below with your immediate boss in mind.
(The person/manager you turn to in your daily work.)
The further to the right you put your response tick, the more you agree with the statement.
Circle the appropriate number.

A. My boss is available when I need him/her

Totally disagree 1 2 3 4 5 6 Totally agree

B. My boss is good at making us interested and committed to our work

Totally disagree 1 2 3 4 5 6 Totally agree

C. My boss helps us divide our work in a fair way

Totally disagree 1 2 3 4 5 6 Totally agree

D. My boss takes things up for discussion in my work group before making important decisions

Totally disagree 1 2 3 4 5 6 Totally agree

E. When necessary, my boss is able to make his/her own decisions

Totally disagree 1 2 3 4 5 6 Totally agree

F. My boss makes sure that information on the operation's goals and visions is available to my work group

Totally disagree 1 2 3 4 5 6 Totally agree

To allow us to use the information you have provided in the questionnaire you must sign the attached consent form and deliver/submit it together with the completed questionnaire.
All the information you have provided will be coded, and the results will not be traceable back to you.

Thank you for taking the time to answer this questionnaire!

Pre-shift questionnaire for acute respiratory and skin symptoms

Date ☐☐ / ☐☐ / ☐☐☐☐ Time ☐☐:☐☐

1. Have you been at work during the past week? ☐ NO ☐ YES

2. Did you suffer from a cold in the last week? ☐ NO ☐ YES

3. Do you have any of the following symptoms **right now**?

Grade the symptoms (circle the appropiate number) on a scale from 0 (no symptoms) to 4 (severe symptoms):

Eye symptoms

Itching

No symptoms 0 1 2 3 4 Severe symptoms

Swelling

No symptoms 0 1 2 3 4 Severe symptoms

Discharge

No symptoms 0 1 2 3 4 Severe symptomsr

Nose symptoms

Itching

No symptoms 0 1 2 3 4 Severe symptoms

Sensation of fullness, congestion, or blockage

No symptoms 0 1 2 3 4 Severe symptomsr

Sneezing

No symptoms 0 1 2 3 4 Severe symptoms

Discharge or runny nose

No symptoms 0 1 2 3 4 Severe symptoms

Respiratory symptoms

Cough

No symptoms	0	1	2	3	4	Severe symptoms

Wheeze (whistling in your chest)

No symptoms	0	1	2	3	4	Severe symptoms

Mucus which is difficult to cough up

No symptoms	0	1	2	3	4	Severe symptoms

Shortness of breath or chest tightness

No symptoms	0	1	2	3	4	Severe symptoms

Skin symptoms

Itchy wheals (urticaria) appearing and disappearing rapidly (within hours) on your hands, wrists or forearms

No symptoms	0	1	2	3	4	Severe symptoms

Post-shift questionnaire for acute respiratory and skin symptoms

Dato __ __ / __ __ / __ __ __ __ Klokkeslett __ __ : __ __

1. In which department did you work today? (Select as many alternatives as apply)

- ☐ Slaughterhouse
- ☐ Filleting
- ☐ Other processing besides filleting
- ☐ Cleaning
- ☐ Packing/freezer room/warehouse
- ☐ Maintenance of machinery/production equipment
- ☐ Administration/office
- ☐ Other

2. Did you experience any of the following symptoms **during your working day**?
Grade the symptoms (circle the appropiate number) on a scale from 0 (no symptoms) to 4 (severe symptoms):

Eye symptoms

Itching

No symptoms 0 1 2 3 4 Severe symptoms

Swelling

No symptoms 0 1 2 3 4 Severe symptoms

Discharge

No symptoms 0 1 2 3 4 Severe symptoms

Nose symptoms

Itching

No symptoms	0	1	2	3	4	Severe symptoms

Sensation of fullness, congestion, or blockage

No symptoms	0	1	2	3	4	Severe symptoms

Sneezing

No symptoms	0	1	2	3	4	Severe symptoms

Discharge or runny nose

No symptoms	0	1	2	3	4	Severe symptoms

Respiratory symptoms

Cough

No symptoms	0	1	2	3	4	Severe symptoms

Wheeze (whistling in your chest)

No symptoms	0	1	2	3	4	Severe symptoms

Mucus which is difficult to cough up

No symptoms	0	1	2	3	4	Severe symptoms

Shortness of breath or chest tightness

No symptoms	0	1	2	3	4	Severe symptoms

Skin symptoms

Itchy wheals (urticaria) appearing and disappearing rapidly (within hours) on your hands, wrists or forearms

No symptoms	0	1	2	3	4	Severe symptoms
