# Supplementary material for: Effects of Interventions to Prevent Work-Related Asthma, Allergy, and Other Hypersensitivity Reactions in Norwegian Salmon Industry Workers (SHInE): Protocol for a Pragmatic Allocated Intervention Trial and Related Substudies
Source: JMIR Res Protoc. 2023 Jul 19;12:e48790. doi: 10.2196/48790 (PMC10398556; doi:10.2196/48790)
Supplement: Multimedia Appendix 2 [file resprot_v12i1e48790_app2.docx]

**Table S1.** Timeline of participants and data gathering

TEWL = transepidermal water loss, HECSI = Hand Eczema Severity Index, PEF = peak expiratory flow

* +/- 2 weeks

** as the study is not designed following only the specific cohort that was enrolled at baseline, participants were recruited and enrolled at both T1 and T2

*** basic meaning assessment was done at 1 arbitrary time point during the week.

|  | **STUDY PERIOD** | | | | | |
| --- | --- | --- | --- | --- | --- | --- |
|  | **Enrollment** | **Allocation** | **Post allocation** | | | |
| **Timepoint** | ***Funding***  ***application (-2 years)*** | ***Intervention week***  ***-3- -1 =* T_0_** | ***Intervention week 0 = T_1_*** | ***Intervention weeks 5-8*** | ***Intervention weeks 31-36*** | ***Intervention week 52* = T_2_*** |
| Enrollment plant | x |  |  |  |  |  |
| Eligibility screening plant | x |  |  |  |  |  |
| Informed consent plant | x |  |  |  |  |  |
| Recruiting of participants |  | x | x |  |  | (x) ** |
| Enrollment individual |  |  | x |  |  | (x) ** |
| Eligibility screening individual |  |  | x |  |  | (x) ** |
| Informed consent individual |  |  | x |  |  | (x) ** |
| Allocation of intervention arms |  |  | x |  |  |  |
| **INTERVENTIONS** |  |  |  |  |  |  |
| NZ |  |  |  |  |  |  |
| CS |  |  |  |  |  |  |
| CTR |  |  |  |  |  |  |
| **ASSESSMENTS:** |  |  |  |  |  |  |
| **Exposure data** |  |  |  |  |  |  |
| Personal exposure - (inhalable protein, enzymes, allergens and endotoxins) |  |  | x |  |  | x |
| Area concentration (inhalable protein, enzymes, allergens, endotoxins) |  |  | x |  |  | x |
| Microbiological sampling (bacteria, fungi) |  |  | x |  |  | x |
| **Health examinations** |  |  |  |  |  |  |
| Blood samples (basic*** and cross-shift/cross-week) |  |  | x |  |  | x |
| Skin prick test |  |  | x |  |  | x |
| Spirometry (basic*** and cross-shift/cross-week) |  |  | x |  |  | x |
| Skin examinations (TEWL, HECSI) in selected plants |  |  | x |  |  | x |
| Serial PEF measurements in selected plants |  |  | x |  |  | x |
| **Questionnaires** |  |  |  |  |  |  |
| Demographic and background data |  |  | x |  |  | x |
| Airway symptoms (basic*** and cross-shift/cross-week) |  |  | x |  |  | x |
| Eye symptoms (basic*** and cross-shift/cross-week) |  |  | x |  |  | x |
| Skin symptoms (basic*** and cross-shift/cross-week) |  |  | x |  |  | x |
| Work environment |  |  | x |  |  | x |
| **Background technical data** | x |  | x |  | x | x |
| **Midterm visit** |  |  |  |  | x |  |
