# Supplementary material for: Effects of Interventions to Prevent Work-Related Asthma, Allergy, and Other Hypersensitivity Reactions in Norwegian Salmon Industry Workers (SHInE): Protocol for a Pragmatic Allocated Intervention Trial and Related Substudies
Source: JMIR Res Protoc. 2023 Jul 19;12:e48790. doi: 10.2196/48790 (PMC10398556; doi:10.2196/48790)
Supplement: Multimedia Appendix 3 [file resprot_v12i1e48790_app3.pdf]

Forskningsrådet

Assessment of grant application submitted to the Research Council of Norway

Grant application

Project number	302902
Project title	Effects of interventions to prevent work-related asthma, allergy and other hypersensitivity reactions in Norwegian salmon industry workers
Project manager	Bang, Berit E.
Project owner	UNIVERSITETSSYKEHUSET NORD-NORGE HF
Application type	Researcher Project /
Programme/Activity	Velferd, arbeid og migrasjon
Case officer	Alexandra Bjørk-Skaflestad

Confirmation

By completing and submitting this form, I / we confirm the following (applies for the individual referee or the referee panel):

- I/We have no conflicts of interest that would prevent me/us from conducting this assessment. See Regulations on Impartiality and Confidence in the Research Council of Norway.	Yes
- I/We have read and understood both the criteria I/we have been asked to use for assessing the application and the description of the scale of marks. The scale of marks is to be applied as an absolute scale, i.e. marks are to be determined for each grant application independently and not relative to other applications that the panel/referee is assessing.	Yes
- I/We understand and accept the guidelines for assessing applications for the Research Council of Norway. See Guidelines for referees/panels who assess applications for the Research Council of Norway.	Yes
- I am/We are qualified to conduct this assessment.	Yes

Summary of marks

Criterion	Mark
Excellence	6
Impact	6
Implementation	5
Overall assessment of the referee/panel	6

Criteria

Excellence

.

The extent to which the proposed work is ambitious, novel, and goes beyond the state of the art
* Scientific creativity and originality.
* Novelty and boldness of hypotheses or research questions.
* Potential for development of new knowledge beyond the current state of the art, including significant theoretical, methodological, experimental or empirical advancement.

The quality of the proposed R&D activities
* Quality of the research questions, hypotheses and project objectives, and the extent to which they are clearly and adequately specified.
* Credibility and appropriateness of the theoretical approach, research design and use of scientific methods. Appropriate consideration of interdisciplinary approaches.
* Where relevant, the extent to which appropriate consideration has been given to ethical issues, safety issues, gender dimension in research content, and appropriate use of stakeholder/user knowledge.

This excellent proposal focuses on testing intervention programmes to reduce exposure to bioactive agents at the workplace in the salmon industry. This is a multicentre and multidisciplinary study involving intervention, and the evaluation of interventional measures, and includes the characterisation of bioaerosols and their potential health hazards by detection of the exposure-response relationship. This project has several novel aspects: evaluation of intervention measures, personalised exposure assessment and a multicentre and multidisciplinary approach, which increases the capacity to clarify this important problem of the salmon industry in Norway, and in addition to suggest intervention measures to reduce exposure to bioaerosols in general.

The hypotheses are clearly stated and research questions are well specified and structured into work packages. This project is likely to generate new knowledge, which can be specifically used in fishery industries and also in areas of increased exposure to bioaerosols. Overall this proposal is excellent.

Selected mark : 6 - Excellent

The proposal successfully addresses all relevant aspects of the criterion. Only minor shortcomings are present.

Impact

.

Potential impact of the proposed research
* The extent to which the planned outputs of the project address important present and/or future scientific challenges.
* If relevant with respect to the project objectives, the extent to which the planned outputs will address UN Sustainable development goals or other important present and/or future societal challenges.
* The extent to which the potential impacts are clearly formulated and plausible.

Communication and exploitation
* Quality and scope of communication and engagement activities with different target audiences, including relevant stakeholders/users.

The proposed study design is excellent with only minor shortcomings (low numbers of included workers for personalised exposure assessment, for instance). This project will have a strong link with end users starting already with the planning phase of the project. The activities to increase the impact on target groups and end users, the representatives of the fishery companies, and local authorities are listed in detail and are feasible and convincing. The planned output of the project has the high potential to answer current scientific challenges and to develop modified strategies for the prevention of job-related allergies and respiratory diseases. The output specifically addresses UN sustainable development goals. These potential impacts are plausible and clearly presented.

A clear and detailed communication plan with different target groups, end users and stakeholders is described.

Selected mark : 6 - Excellent
The proposal successfully addresses all relevant aspects of the criterion. Only minor shortcomings are present.

Implementation

.

The quality of the project manager and project group
* The extent to which the project manager has relevant expertise and experience, and demonstrated ability to perform high-quality research (as appropriate to the career stage).
* The degree of complementarity of the participants and the extent to which the project group as a whole assembles the necessary expertise needed to undertake the research effectively, and provides added value.

The quality of the project organization and management
* Effectiveness of the work plan, including the extent to which resources assigned to work packages are aligned with project objectives and deliverables.
* Appropriateness of the allocation of tasks, ensuring that all participants have a valid role and adequate resources in the project to fulfil that role.
* Appropriateness of the proposed management structures and governance.

The study design is clear and includes a series of measurement and then intervention components which make the sensible use of this unique sampling frame. There were some shortcomings of the approaches being employed - for example sampling to measure exposure from the masks of the workers (i.e. confounded by wearing the masks) and the likely confounded nature of the workforce - i.e. a self selected pool which have other characteristics which will confound health outcome associations - however the panel were impressed by the overall concept and the opportunity to address a question relevant to Norway specifically.

The project manager has shown her very good expertise in the launching and coordination of large research activities. While she has a good publication record within the last ten years the publication track record of the members of the project group is heterogeneous: Several have only few publications in peer–reviewed journals so far, other project partners are very well-known and distinguished scientists. This combination will help the less experienced to write and publish papers in high-ranked scientific journals. The project group as a whole assembles well-experienced researchers in occupational hygiene and exposure assessment, which is required to make this proposal a success.
The work plan is very well structured and details on each of the underlying research questions are given. The experts of different fields are appropriately allocated to the different tasks and research questions.

Selected mark : 5 - Very good
The proposal addresses the criterion very well. A small number of shortcomings are present.

Overall assessment of the referee/panel

.

Overall assessment of the referee/panel based, on the criteria Excellence, Impact and Implementation.

Overall, this is an excellent proposal, which cannot be performed better in any other country, because the salmon industry is more common in Norway than in any other place of the world. This project is extremely necessary to improve health of workers in the salmon industry, which is essential for the economic growth of the entire country. The multicentre and multidisciplinary project will contribute to capacity building in occupational hygiene and medicine in Norway. This proposal has several aspects of novelty: the intervention, which goes beyond the pure observational aspects of many other similar studies, however, more information on the intervention programme would have been useful to read.

Nevertheless, part of the interventional strategies will be derived only after detailed exposure assessment within this project. One key question which is not really articulated is the number of workers this is likely to affect and, within that pool, the comparative frequencies of the health outcomes of interest here. There is then the question as to the confounded nature of this form of employment and how this will be cut through in order to understand better the likelihood of the health outcomes of interest being specifically related to the exposures of interest. Multiple sites (with different demographics), interventions and other approaches may be need to get a feel for the likely existence of causal relationships in this otherwise observational work.

As mentioned, there are then some queries about the nature of the work itself - for example the use of MS approaches to examine the masks/filters of staff is an interesting idea - though potentially confounded by the wearing of the mask itself. To this end, the nature of the sampling frames (i.e. getting samples from the workspace) and information from workers on the procedures used to avoid risk may actually be more compelling. With this the intervention approaches are also more exciting. Again - assessments such as those in WP4 appear excellent and likely to contribute, but information on the feasibility of these sampling efforts would be welcome. The project group as a whole assemblies all the needed multidisciplinary knowledge.

The panel were keen to stress that this application was a good design and was likely to make excellent use of an important epidemiological opportunity. There were some limitations to the overall approach, however the combination of research group, relatively simple and well designed approach, pertinent measurements and analyses and in a field with real potential scientific and public health impact was attractive. This proposal scored well overall.

Selected mark : 6 - Excellent
The proposal is of excellent quality, and of a very high international standard. All relevant aspects of the criteria are successfully addressed. Only minor shortcomings are present.

Special points to consider

Comments to special points to consider
